# Attempted use of PACE for riboswitch discovery generates three new translational theophylline riboswitch side products

**DOI:** 10.1186/s13104-018-3965-6

**Published:** 2018-12-05

**Authors:** Zachary M. Shaver, Stephanie S. Bent, Steven R. Bilby, Michael Brown, Anna Buser, Itzayana G. Cuellar, Athena J. Davis, Lindsay Doolan, Fatima C. Enriquez, Autumn Estrada, Shelby Herner, J. Cody Herron, Andrew M. Hunn, Madison Hunter, Hartlee Johnston, Owen Koucky, Christian C. Mackley, Dylan Maghini, Devin Mattoon, Haden T. McDonald, Hannah Sinks, Austin J. Sprague, David Sullivan, Altan Tutar, Avery Umphreys, Chris Watson, Daniel Zweerink, Laurie J. Heyer, Jeffrey L. Poet, Todd T. Eckdahl, A. Malcolm Campbell

**Affiliations:** 1 Department of Biology, Davidson College, Davidson, USA; 2 Department of Biology, Missouri Western State University, Saint Joseph, USA; 3 Department of Computer Science, Math, and Physics, Missouri Western State University, Saint Joseph, USA; 4 Department of Math and Computer Science, Davidson College, Davidson, USA

**Keywords:** Phage-assisted continuous evolution (PACE), Riboswitch, Synthetic biology, Metabolic engineering, Theophylline, Xanthine, M13

## Abstract

**Objective:**

The purpose of this project was to use an in vivo method to discover riboswitches that are activated by new ligands. We employed phage-assisted continuous evolution (PACE) to evolve new riboswitches in vivo. We started with one translational riboswitch and one transcriptional riboswitch, both of which were activated by theophylline. We used xanthine as the new target ligand during positive selection followed by negative selection using theophylline. The goal was to generate very large M13 phage populations that contained unknown mutations, some of which would result in new aptamer specificity. We discovered side products of three new theophylline translational riboswitches with different levels of protein production.

**Results:**

We used next generation sequencing to identify M13 phage that carried riboswitch mutations. We cloned and characterized the most abundant riboswitch mutants and discovered three variants that produce different levels of translational output while retaining their theophylline specificity. Although we were unable to demonstrate evolution of new riboswitch ligand specificity using PACE, we recommend careful design of recombinant M13 phage to avoid evolution of “cheaters” that short circuit the intended selection pressure.

**Electronic supplementary material:**

The online version of this article (10.1186/s13104-018-3965-6) contains supplementary material, which is available to authorized users.

## Introduction

An important goal of microbial metabolic engineering is the development of improved methods for the integration of host and orthogonal metabolism [1–5]. Our research group developed a generalized method called programmed evolution for optimizing orthogonal metabolic engineering [6]. The key to programmed evolution is a fitness module containing a riboswitch (RS) that is regulated by the desired metabolic product. The extent to which programmed evolution can be applied is limited by the availability of RSs. RSs discovered in vitro often do not predictably control gene expression in vivo [7]. Rational approaches to RS discovery exist but they do not facilitate exhaustive sequence space searches [8]. We hypothesized that phage-assisted continuous evolution (PACE) would have advantages for RS discovery [9–11]. PACE relies on sustained phage populations and induced mutations coupled with positive and negative selection of M13 phage grown in chemostats.

**Fig. 1:**
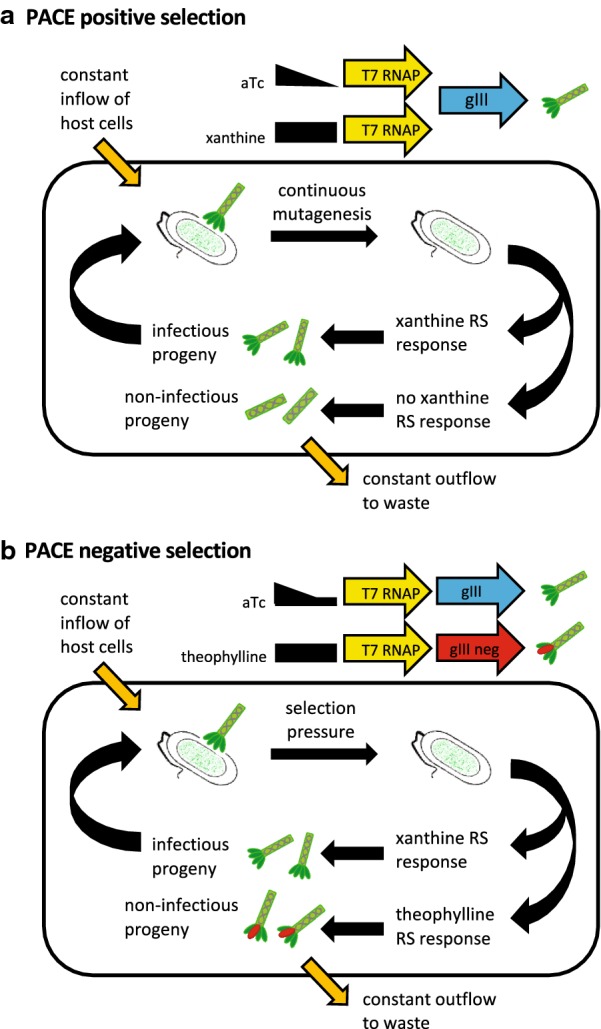
PACE experimental design for the discovery of new RSs. **a** During positive selection, anhydrotetracycline (aTc) concentration is reduced while the new target ligand xanthine concentration remained constant. Mutagenesis cassette is induced using arabinose. The chemostat replenishes growth media and uninfected *E. coli*. **b** Negative selection employed a dominant gIII negative allele (gIII neg) induced by a steady concentration of theophylline to select against theophylline-specific riboswitches while gradually reducing aTc to maintain phage with xanthine-specific riboswitches

The experimental design we developed was intended to be a proof-of-concept for using PACE to discover RSs with new ligand specificity (Fig. 1). Our goal was to evolve xanthine-specific transcriptional and translational RSs from existing RSs that bind theophylline. Our attempt to evolve new xanthine RSs from theophylline RSs did not succeed. The RSs accumulated many mutations after positive selection but negative selection did not occur. This report provides details of our experiments and their limitations so that others might build on our work to develop PACE for RS discovery. We also report side products in the form of thee new theophylline-specific translational RS with different translational efficiencies and induction ratios.

## Main text

Our adaptation of PACE placed a RS between the gene three (gIII) promoter (PIII) and the T7 RNA polymerase (RNAP) gene so that the RS was the target of directed evolution (Additional file 1: Figure S1 in PACE Additional Information). We chose RS-C from Topp et al. as the translational RS, and RS10 from Wachsmuth et al. as the transcriptional riboswitch [12, 13]. RS-C blocks production of T7 RNA polymerase unless theophylline binds to its aptamer and alters its folding so that the RBS is available for the initiation of translation. Binding of theophylline to RS10 disrupts the formation of a stem and loop structure required for the termination of transcription. We conducted three simultaneous PACE experiments in our chemostat apparatus (see details in Additional file 2: PACE Chemostat Manual 2016). One PACE chemostat contained phages that only carried RS-C, a second chemostat contained only phages with RS10, and a third contained both RSs. The objective for positive selection was to evolve a phage variant that contained a xanthine-activated RS. The experiment began with a 24 h gradual increase in positive selection for RSs that respond to xanthine instead of theophylline. The inclusion of anhydrotetracycline (aTc) in the experiment is for T7 RNAP-driven protein three (pIII) production initially until xanthine-responsive RSs arise and accumulate, after which aTc levels were gradually reduced (see Fig. 1 and Additional file 1: Appendix A in PACE Additional Information). During positive selection, we used arabinose to induce a host cell mutagenesis gene expression cassette that increases the rate of DNA mutations among phage progeny. We successfully measured green fluorescent protein (GFP) fluorescence as an indirect measure of pIII production, and used polymerase chain reaction (PCR) to confirm the persistence of M13 genomes in the chemostats (see Additional file 1: Methods in PACE Additional Information).

The objective of negative selection was to eliminate phages with RSs that are constitutively activated and do not require ligand binding to achieve an ON state and to eliminate phages with RSs that still have theophylline responsiveness. After a short transitional period to purge xanthine from the experiment, we began 16 h of negative selection with a new host strain that lacked the mutagenesis cassette to prevent evolution away from the desired RS outcome. We provided a steady supply of theophylline but gradually reduced the amount of aTc (see Fig. 1 and Additional file 1: Appendix A in PACE Additional Information). Throughout negative selection, we were able to confirm the presence of M13 DNA by PCR, but we failed to detect red fluorescent protein (RFP) as an indirect measure of the gIII neg expression (see Fig. 1 and Additional file 1: Figure S1 in PACE Additional Information). Despite our inability to confirm negative selection, we isolated phage from all three evolving chemostat populations and used PCR to amplify the RS regions of M13.

To determine the level of sequence variation among RSs carried by phage populations after PACE, we used paired-end MiSeq to sequence 3.9 million RSs from the three chemostats. Our sequence analysis revealed a high frequency of two types of RSs that were present at the ends of our three PACE experiments (see Additional file 1: Appendix B in PACE Additional Information). From the two PACE lagoons with RS10, we isolated RS10Δ7, which has a seven nucleotide deletion that disrupts the stem and loop structure required for transcriptional termination. We verified that RS10Δ7 is constitutively activated, which explains how it survived positive selection, but not how it escaped negative selection (see Additional file 1: Figure S2 in PACE Additional Information). In the RS-C experiments, we found multiple RSs that should not have persisted through either positive or negative selection (see Additional file 1: Table S2 in PACE Additional Information). The failure of negative selection was consistent with our inability to detect RFP.

The inability of our PACE experiments to exert positive selection for xanthine riboswitches and negative selection against theophylline-responsive RSs might be explained by an inherent experimental design flaw. There are more ways to evolve a RS that remains in an ON state than to evolve a RS with new ligand specificity. Our evolving populations accumulated “cheaters” during positive and negative selection. Our suggestion for others who attempt PACE for RS evolution is to avoid an experimental strategy in which disabling the DNA of interest is adaptive for the M13 phage under selection. A better strategy would enable simultaneous negative selection against RS OFF states and positive selection for ON states that are dependent on a desired ligand specificity. Positive and negative selection might need to be run longer since the RS target sequence is small compared to the entire M13 phage genome. It might take longer to accumulate sufficient mutations to alter ligand binding.

**Fig. 2:**
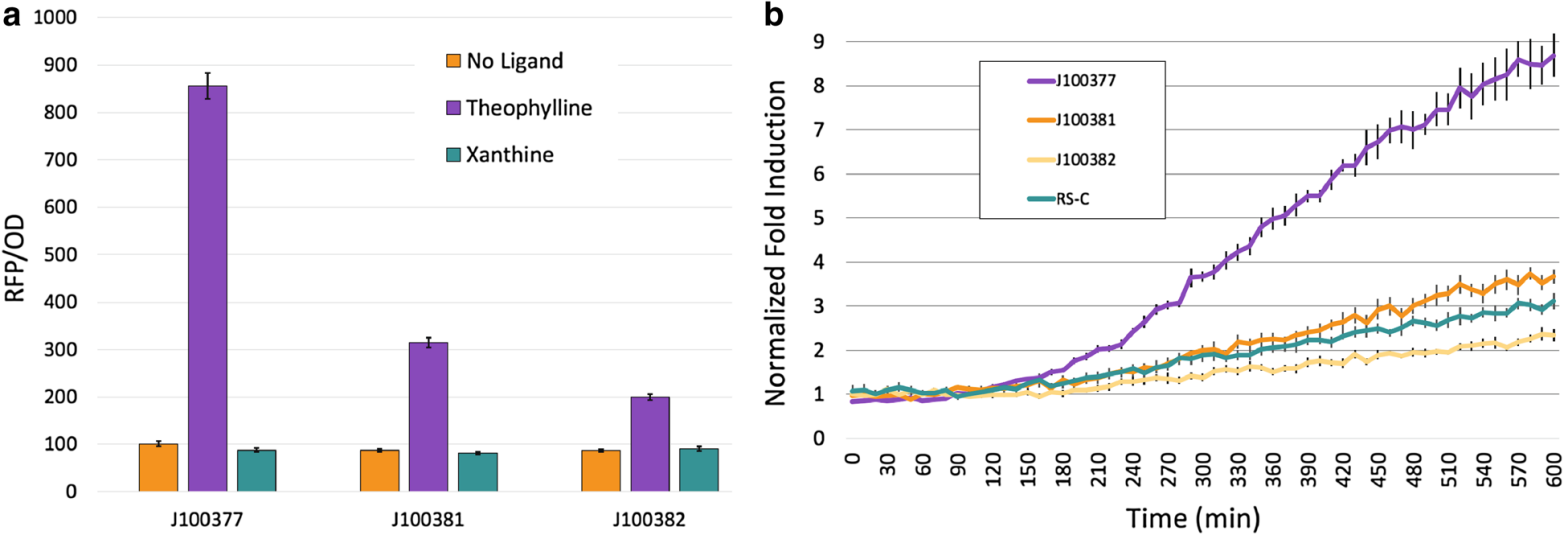
Directed evolution of three new riboswitches. **a** Each new riboswitch was cloned into rClone Red and grown in triplicate over 10 h with one of three treatments as indicated. Each population was measured for RFP and cell density three times to produce an average end point value ± s.e.m. **b** Theophylline-induction values from three independent populations from **a** were normalized by comparing each construct to the fold induction of RS-C in the absence of theophylline. Error bars represent s.e.m

**Fig. 3:**
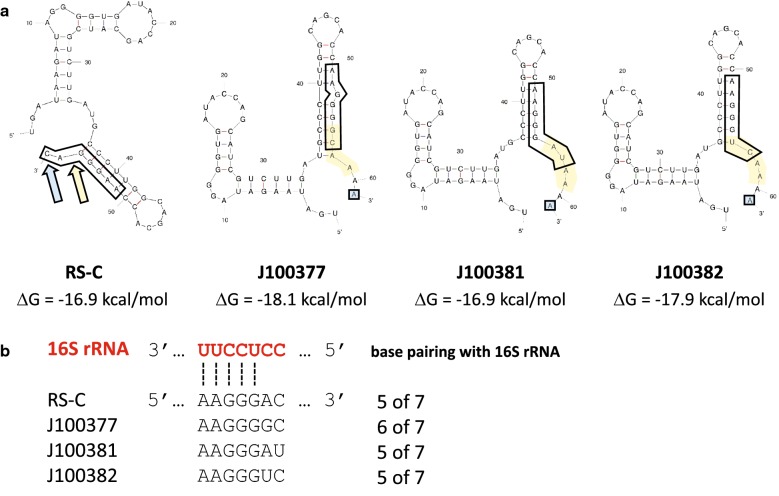
Predicted 2D structures of RS-C and three new riboswitches. **a** The original RS-C sequence (left) was submitted to mFold to produce a predicted 2D structure and quantification of stability (ΔG); the boxed region is the predicted RBS. The three new riboswitch sequences were also evaluated by mFold with RBS denoted by boxed nucleotides. Yellow highlighting shows inserted bases and the blue A within a box indicates a base substitution conserved in all three new riboswitches. **b** RNA base pairing (dashed lines) between ribosome (red) and the complementary anti-parallel  riboswitch sequence (black) for the original RS-C as well as the three new RSs. The number of base pairs between rRNA and RS sequence is shown on the far right

Despite our inability to evolve xanthine-specific RS, we discovered side products in the form of three new theophylline-specific RSs with three levels of ON state translational output (Fig. 2). These new RSs retained their original ligand specificity and shared similar mutations in and near their RBSs (Fig. 3). Each of the three new RS carried the same single base change plus unique insertions of 4 bases adjacent to the RBS. To explain our experimentally observed differences in translational output, we used mFold to predict secondary structures of the new RSs and compared them to original RS-C. Each of the three new RSs adopts a stem and loop structure of similar stability to the one found in RS-C, which supports the measurement of their OFF states. To assess ON states, we analyzed the ability of each of the RS RBSs to base pair with the 3′ end of 16S rRNA. The new RS J100377 can form 6 base pairs, whereas the other three RSs form only 5. These results are consistent with our observation of higher expression from the J100377 RS than the other RSs.

## Limitations

We acknowledge several limitations to our contribution to the synthetic biology literature of three new theophylline-specific translational RSs and of a protocol for using PACE to evolve RSs. Positive selection during our PACE experiments worked to some degree, but was confounded by the fitness advantage of a “cheater” with an easily-produced deletion that enabled constitutive pIII production. Negative selection failed during our PACE experiments, perhaps because of undetected mutations in gIII neg. We acknowledge that it might not be possible to evolve theophylline RSs into xanthine RSs with PACE. This shortcoming could be addressed by using other ligands or other RS starting points. The paper from which we chose RS-C includes several RSs that show higher levels of reporter gene expression than J100377, J100381, and J100382, and one with a larger induction ratio [12]. Translational RSs with similar characteristics can also be found in other publications [14–16]. We have not measured the theophylline dose–response or explored their modularity with regard to various promoter strengths and reporter genes.

## Additional files

**Additional file 1.** Contains additional information that will help users fully understand the research project described by this manuscript.

**Additional file 2.** Chemostat Manual: user manual for building a chemostat for phage-assisted continuous evolution (PACE). This is a detailed explanation of how to build your own chemostats for PACE experiments. This document also contains all parts numbers and suppliers so users can purchase the same materials we used.

## Abbreviations

RS: riboswitch
PACE: phage-assisted continuous evolution
PIII: promoter to gene three (gIII)
gIII: gene three
pIII: protein three
RNAP: RNA polymerase
aTc: anhydrotetracycline
GFP: green fluorescent protein
RS10Δ7: RS10 with a 7 base pair deletion
PCR: polymerase chain reaction
RFP: red fluorescent protein
