# Supplementary material for: Attempted use of PACE for riboswitch discovery generates three new translational theophylline riboswitch side products
Source: BMC Res Notes. 2018 Dec 5;11:861. doi: 10.1186/s13104-018-3965-6 (PMC6280357; doi:10.1186/s13104-018-3965-6)
Supplement: Supplementary file 1 — Additional file 1. Contains additional information that will help users fully understand the research project described by this manuscript. [file 13104_2018_3965_MOESM1_ESM.docx]

**Additional Figures, Tables, Data, Methods and Appendices**


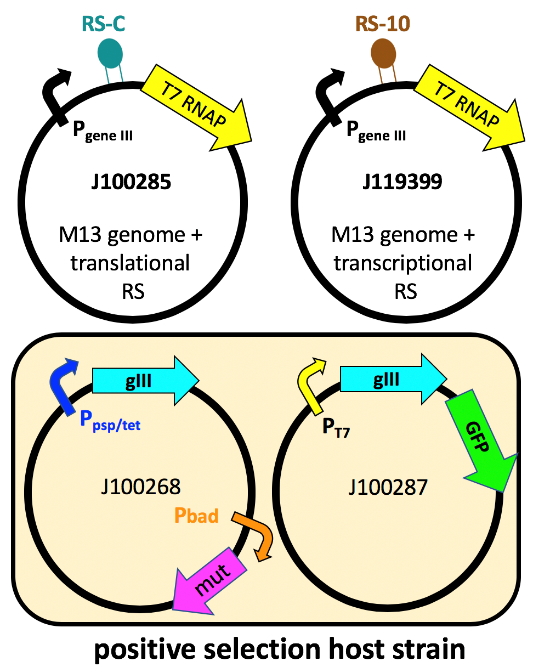

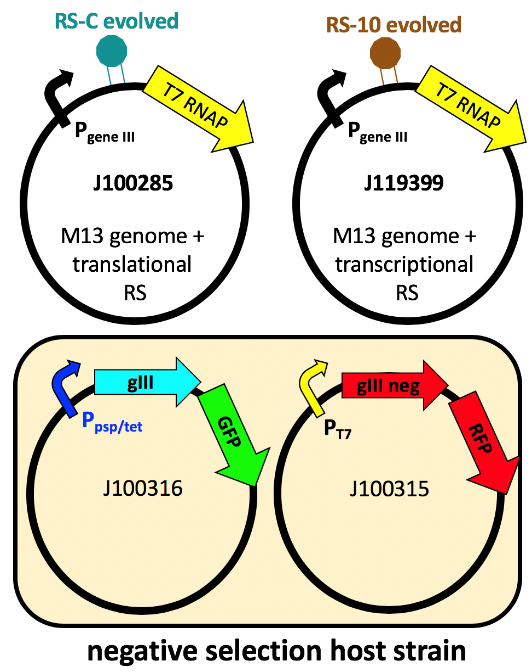
  **A** **B**

**Additional file 1: Figure S1.** **Genotypes of host strains and recombinant M13 phage.** A) Positive selection host E. coli JM109 containing two plasmids. Plasmid J100268 has a hybrid promoter upstream of a wild type (wt) gene III. This hybrid promoter requires M13 infection plus anhydrotetracycline (aTc) to induce transcription. It also carries a mutagenesis cassette (mut) under the control of the Pbad promoter, which is induced with arabinose. Plasmid J100287 uses a T7 promoter to transcribe an operon that includes a wt gene III and a GFP reporter gene. Recombinant M13 phage have their gene III replaced with a riboswitch upstream of the T7 RNA polymerase (RNAP) gene. M13 genome J100285 contains riboswitch-C (RS-C), which binds to theophylline to permit translation of a downstream T7 RNA polymerase (RNAP) ORF. M13 genome J119399 carries the transcriptional riboswitch 10 (RS10), which binds to theophylline to permit transcription of a downstream T7 RNAP gene. B) The phage at the top are the same as in panel A except their riboswitches may have mutated and evolved. The negative selection host strain JM109 carries two plasmids. J100316 carries a hybrid promoter upstream of an operon containing wt gene III and a GFP reporter gene. J100315 carries a T7 promoter for transcription of an operon consisting of the gene III dominant negative allele (g III neg) and a RFP reporter gene.


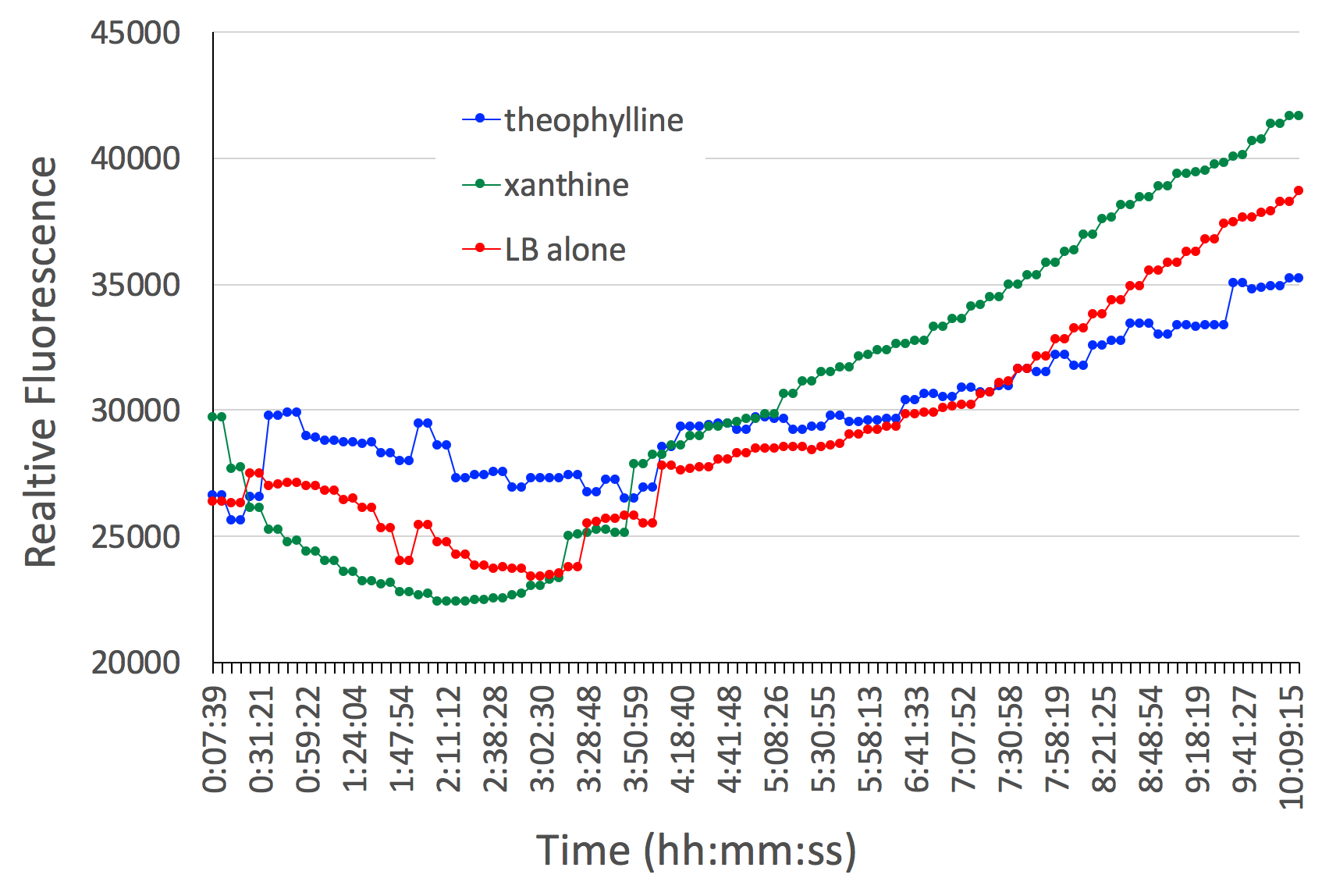


**Additional file 1: Figure S2. Common seven base deletion within the aptamer breaks riboswitch.** A common variant of riboswitch 10 (RS10) after negative selection was a seven base pair deletion within the riboswitch. This sequence was cloned into rClone Red (J119384; <http://parts.igem.org/wiki/index.php/Part:BBa_J119384>) and tested for RFP expression in JM109 cells over 10 hours of growth at 37º C in LB ampicillin broth alone or with xanthine or theophylline added (2 µM). The expression level of the RFP reporter gene was similar in all conditions, indicating positive selection worked but negative selection failed to purge riboswitches broken in the “on” state.

**Additional file 1: Methods**

*Phage Assisted Continuous Evolution (PACE)*

Plasmids used for PACE were obtained from David Liu’s laboratory at Harvard University. All oligonucleotides were ordered from Integrated DNA Technologies. All ligations were performed with T4 DNA ligase (New England Biolabs). The PACE experiment included three phases: positive selection, a transition phase, and negative selection (see Appendix A for detailed instructions). The experiment included three chemostats called “lagoons” where the phage infect bacteria and replicate. Details of how to build a chemostat are in the supplemental Chemostat Manual. The first lagoon was supplied with a J100285 phage population containing RS-C, the second with a J119399 phage population containing RS10, and the third with both J100285 and J119399 populations. Throughout positive selection, a syringe pump (Cole Parmer) was used to provide each lagoon with anhydrotetracyclince (aTc) and increasing concentrations of xanthine to select for riboswitches that can bind to xanthine. During the transition phase, the host cells were switched and the lagoons were provided with theophylline. During negative selection, the lagoons were provided with theophylline and decreasing concentrations of aTc to select for phage containing riboswitches specific to xanthine. aTc activates the psp/tet promoter in J100268 and J100316 to allow for the production of protein III, which enables phage containing the correct riboswitch to reproduce during negative selection.

*PCR of RS after PACE*

PCR was conducted using Gotaq G2 Green Master Mix (Promega): 95°C, 2 min, (95°C, 30 sec, 58°C, 30 sec, 72°C, 10 sec) 30 cycles. The barcodes/adapters were supplied by the company who performed MiSeq next generation sequencing on the amplicons. RS-C amplicons were 190 bp whereas RS10 amplicons were 209 bp.

FW Primer: TCGTCGGCAGCGTCAGATGTGTATAAGAGACAGGCAGTTTCATTTGATGCTCG

REV Primer: GTCTCGTGGGCTCGGAGATGTGTATAAGAGACAGGGTCAGCCAGAGTGTTGAAC

underline = MiSeq adapters added to 5’ end of both primers

*Evaluation of Mutant Sequences*

A python program (see Appendix B) was used to organize in order of frequency DNA sequences of phage taken from the final samples of each PACE lagoon. Ten of the most prevalent RS-C mutants were selected for testing and labeled #1-10 (Table 2).

*Insertion of Mutants into rClone Red Plasmids*

The mutant sequences were separated into smaller strands and ordered from Integrated DNA Technologies. The oligonucleotides were annealed by boiling and cooling to form complete riboswitch sequences. Golden gate assembly (GGA) was performed in a thermal cycler (Techne Prime) to insert each riboswitch into the rClone Red plasmid (J119384). The plasmids were transformed into Zippy Mix-and-Go JM109 competent cells (Zymo Research), which were grown on LB agar plates with 100 µg/mL ampicillin. Cells containing rClone Red with each riboswitch mutant were miniprepped and sequenced (Eurofins). Freezer stocks were made of cells containing plasmids with the correct riboswitch sequences. Only sequence verified stocks were used for experimentation.

*RFP Fluorescence Assay*

Plasmids containing mutant riboswitch sequences were grown overnight in LB ampicillin media. 50 µL of each culture was mixed with 150 µL of media containing no ligand, 2 µM theophylline, or 2 µM xanthine in a 96-well plate. Red fluorescence (excitation: 585 nm, emission: 615 nm) and optical density at 600 nm were measured over a 10 hour period in a Synergy H1 microplate reader (BioTek), with constant shaking and measurements taken every 10 minutes. Plasmids containing rClone Red with the original RS-C sequence (J100261) were used as the positive control, and plasmids containing the unmodified rClone Red cassette (J119384) were used as the negative control. Biological and technical triplicates were used.

**Additional file 1: Table S1** Host strains, plasmids and recombinant M13 phage details.

| **Component** | **Part numbers** | **Plasmid details** |
| --- | --- | --- |
| modified M13 phage with  translational riboswitch C | J100285 (see SOM Figure 1) | Ori = F1 kan^R^ |
| modified M13 phage with  transcriptional riboswitch 10 | J119399 (see SOM Figure 1) | Ori = F1 kanamycin resistant |
| positive selection host  strain JM109 | J100268  J100287  (see SOM Figure 1) | Ori = CloDf13; chlor^R^  Ori = SC101; carb^R^ |
| negative selection host  strain JM109 | J100316  J100315  (see SOM Figure 1) | Ori = CloDf13; chlor^R^  Ori = SC101; carb^R^ |

**Additional file 1: Table S2.** Wild-type RS-C, with RBS highlighted blue, and 10 abundant mutations (yellow) after negative selection.

| RS-C Mutant | Identifying  Number | Sequence (5’ -> 3’) |
| --- | --- | --- |
| WT | J100261 | TGATAAGATAGGGGTGATACCAGCATCGTCTTGATGCCCTTGGCAGCACCAAGGGAC |
| #1 | J100375 | TGATAAGATAGGGGTGACCCCAGCATCGTCTTGATGCCTTTGGCGGCACCAAGGGAC |
| #2 | J100376 | TGATAAGATAGGGGTGACCCCAGCATCGTCTTGATGCCGTTGGCGGCACCAAGGGAC |
| #3 | J100377 | TGATAAGATAGGGGTGATACCAGCATCGTCTTGATGCCCTTGGCAGCACCAAGGGGCAAAA |
| #4 | J100378 | TGATAAGATAGGGGTGAACCCAGCATCGTCTTGATGCCTTTGGCCGCGCCAAGGGAC |
| #5 | J100379 | TGATAAGGTAGGGGTGACCCCAGCATCGTCTTGATGCCTTTGGCGGCACCAAGGGAC |
| #6 | J100380 | TGATAAGATAGGGGTGAACCCAGCATCGTCTTGATGCCTTTGGCCGCACCAAGGGAC |
| #7 | J100381 | TGATAAGATAGGGGTGATACCAGCATCGTCTTGATGCCCTTGGCAGCACCAAGGGATAAAA |
| #8 | J100382 | TGATAAGATAGGGGTGATACCAGCATCGTCTTGATGCCCTTGGCAGCACCAAGGGTCAAAA |
| #9 | J100383 | GTGATACCAGCATCGTCTTGATGCCCTTGGCAGCACCAAGGGAC |
| #10 | J100384 | TGATAAGATAGGGGTGACACCAGCATCGTCTTGATGCCTTTGGCGGCACCAAGGGAC |

**Additional file 1: Appendix A: PACE Detailed Protocol**

Reagents, supplier, product number

ampicillin (Fisher; BP1760-25)

arabinose (Sigma Aldrich; A3256-100G)

anhydrotetracycline (TaKaRa Bio Inc.; 631310)

carboxicillin (ThermoFisher; 10177012

chloramphenicol (Fisher; BP904-100)

kanamycin (Fisher; BP906-5)

LB broth mixture (Acumedia Manufacturers, Inc.; 7290B)

sodium hydroxide (Fisher; S318-100)

theophylline (Sigma-Aldrich; T1633-100G)

xanthine (Sigma-Aldrich; X0626-25G)

**Syringe solutions**

**positive selection** reduces aTc gradually, increase selection pressure on RS, dripped into ~30 mL lagoon at 1 mL/hr.

P1 40 µg/mL aTc 1-2 hours without phage, 5 hours with phage

0.5 M arabinose

30 mg/mL xanthine

1 M NaOH

P2 20 µg/mL aTc 6.25 hours

0.5 M arabinose

30 mg/mL xanthine

1 M NaOH

P3 2 µg/mL aTc 7 hours

0.5 M arabinose

30 mg/mL xanthine

1 M NaOH

P4 0 µg/mL aTc 6.5 hours

0.5 M arabinose

30 mg/mL xanthine

**transition period** starts with high aTc and introduces theophylline, no selection pressure on RS, dripped into ~30 mL lagoon at 1 mL/hr.

T1 100 µg/mL aTc 15 minutes

100 mM NaOH

36 mg/mL theophylline

**negative selection** reduces aTc gradually, increase selection pressure on RS, dripped into ~30 mL lagoon at 1 mL/hr.

N1 40 µg/mL aTc 5 hours

100 mM NaOH

36 mg/mL theophylline

N2 20 µg/mL aTc 5 hours

100 mM NaOH

36 mg/mL theophylline

N3 2 µg/mL aTc 6 hours

100 mM NaOH

36 mg/mL theophylline

*autoclave 8 beakers for syringe solutions

*autoclave 15-20 L of LB for media flow

*prepare 30 sterile 1.5 mL centrifuge tubes containing 300 µL glycerol for sampling, label well

1. Vortex these solutions until everything is in solution
2. Heating P1-P4 syringes will turn the solution brown (unsure if this is detrimental)
3. Use 0.2 µL syringe filters and a BD 5 mL syringe to filter the syringe solutions into new, sterile 50 mL conical tubes
   1. For P1-P4, you may have to use a 0.45 µL filter first to filter out any particulates that did not fully go into solution
4. Grow host cells overnight in 50 mL glass bottles of LB media and necessary antibiotics. Use a disposable inoculating loop (yellow) to sample necessary cells from the freezer stock or plates with colonies, slosh loop in prepared glass bottle and dispose of it after use.
   1. Positive selection: J100268/J100287
   2. Negative selection: J100315/J100316
5. Produce necessary phage
   1. J100285 (translational riboswitch)
   2. J100399 (transcriptional riboswitch)
6. A few hours before intended start time of PACE, turn on the hotplate stirrers and let the water surrounding the lagoon bottles and the reservoir bottle reach a temperature of 36.5-38 °C
7. About an half hour before syringes loading, retrieve necessary syringe solution, and make sure that everything is in solution. If not, put in water bath for about 20 minutes and vortex it until everything is dissolved.
8. Transfer glass bottle of host cells into the reservoir position using sterilized (by dipping in EtOH and flaming) forceps to transfers the needles attached to the red bottle cap into the bottle containing the host cells
9. Retrieve **P1** sterilized syringe solution and pour the entire solution into an autoclaved, sterilized beaker
10. Retrieve 3 sterile BD 60 mL syringes and expel all of the air out of the syringe by pushing the plunger down as far as you can
11. Dip syringe in beaker and pull up indicated volume of solution (in this case 12 mL)
    1. flip the syringe so it stands up on the plunger, expel any air by pushing the plunger until there is no air left
    2. Repeat for the rest of syringes
12. Secure syringes into syringe pump so that the top of the plunger and the top of the barrel are locked into place between two metal pieces
13. Set rate as desired (1 mL/hr)
14. Let run for 1hr 15 min - 2hrs, without phage
15. Take a ~1 mL sample from the tubing between each lagoon and the waste, put in sterile 1.5 mL centrifuge tubes with 300 µL of glycerol and store in -20°C freezer
16. Add necessary phage by opening the lids of each lagoon and pipetting 500 µL of indicated phage
17. Turn off pump between each syringe change
18. Leave **P1** syringes loaded
    1. run for 5 hrs
    2. take sample
19. Repeat syringe preparations and load **P2** syringes
    1. run for 6.25 hrs (6 hrs and 15 mins)
    2. take sample
20. Repeat syringe preparations and load **P3** syringes
    1. run for 7 hrs
    2. take sample
21. Repeat syringe preparations and load **P4** syringes
    1. run for 6.5 hrs (6 hrs and 30 min)
    2. take sample
22. Turn off chemostat
23. Retrieve overnight grown host cells for negative selection and swap that glass bottle with the glass bottle currently in the reservoir, use sterilized forceps to maneuver the needles
24. Repeat syringe preparations and load **T1** syringes
    1. run for 15 mins
    2. take sample
25. Repeat syringe preparations and load **N1** syringes
    1. run for 5 hrs
    2. take sample
26. Repeat syringe preparation and load **N2** syringes
    1. run for 5 hrs
    2. take sample
27. Repeat syringe preparations and load **N3**
    1. run for 6 hrs
    2. take sample

**Additional file 1: Appendix B: Python Program Used to Sort Riboswitch Mutants**

'''

Author : Itzayana Cuellar

Date: 11/25/17

'''

import csv

from graphics import*

def user_input():

'''

produces graphic, user enters required information

argument:

none

return:

file_name = name of file information is to be retrieved from

name_interest = what is the name of the area of interest

position_interest1 = index area of interest begins

position_interest2 = index area of interest ends

output_file = name of output file (to be written)

'''

#set up window

win = GraphWin('Riboswitch analysis', 500,500)

win.setCoords(0,0,100,100)

win.setBackground('cornsilk')

#set up entry box to obtain user entered input file name

file_name_box = Entry(Point(50,75),75)

file_name_box.setFill('white')

file_name_box.draw(win)

#set up entry box to obtain area of interest name

name_interest_box = Entry(Point(26,52.5),35)

name_interest_box.setFill('white')

name_interest_box.draw(win)

#set up entry box to obtain begining index of area of interest

position_interest_box1 = Entry(Point(65,52.5),15)

position_interest_box1.setFill('white')

position_interest_box1.draw(win)

#set up entry box to obtain end index of area of interest

position_interest_box2 = Entry(Point(85,52.5),15)

position_interest_box2.setFill('white')

position_interest_box2.draw(win)

#set up entry box to obtain output file name

output_file_box = Entry(Point(50,30),75)

output_file_box.setFill('white')

output_file_box.draw(win)

#set "GO!" box

enter_box = Rectangle(Point(80,10),Point(95,15))

enter_box.setFill('green')

enter_box.draw(win)

#sets up and draws all text boxes above corresponding entry box

window_text1 = Text(Point(50,95),

'Please enter the following information to recieve file')

window_text2 = Text(Point(50,80),

'Name of file')

window_text3 = Text(Point(25,58),

'Area of interest name')

window_text4 = Text(Point(75,58),

'Area of interest location (format = #,#) ')

window_text5 = Text(Point(50,35),

'Output file')

window_text6 = Text(Point(87.5,12), 'Go!')

window_text1.draw(win)

window_text2.draw(win)

window_text3.draw(win)

window_text4.draw(win)

window_text5.draw(win)

window_text6.draw(win)

#get mouse click, if click is in the go box, retrives all

#entry box information

mouse_click = win.getMouse()

TF=True

while TF:

if 80< mouse_click.getX() < 90 and 10 < mouse_click.getY()< 15:

file_name = file_name_box.getText()

file_name = file_name.strip()

name_interest = name_interest_box.getText()

name_interest = name_interest.strip()

position_interest1 = position_interest_box1.getText()

position_interest1 = position_interest1.strip()

position_interest1 = int(position_interest1)

position_interest2 = position_interest_box2.getText()

position_interest2 = position_interest2.strip()

position_interest2 = int(position_interest2)

output_file = output_file_box.getText()

output_file = output_file.strip()

win.close()

#if click is inside "GO!" box, returns file_name,

#name of position of interest, beging and end area indexes

#and output file name

return (file_name,name_interest,

position_interest1,position_interest2, output_file)

else: #wait for another click if the click was not in the GO! button

mouse_click = win.getMouse()

def get_file_info(file_name):

'''

reads file and produes lists of aligned sequences, reference sequences

and sequency frequency

argument:

file_name = user inputed name of file to import from

returns:

name_read = list with name of sequence as a str(number)

aligned_seq = list of all aligned sequences

reference_seq = list of all reference sequences

'''

#open the file indicated by user

with open(file_name, 'rU') as alles_file:

alles_data = csv.reader(alles_file, delimiter = '\t')

#skip the first (header) line

alles_data.next()

#in each row of the file, retrive colums with

# 'aligned sequence', 'reference sequence'

#and sequence frequency

aligned_seq = []

reference_seq = []

seq_frequency = []

#loop to make lists with aligned sequences,

# reference_sequences, and allele frequencies

for sequence in alles_data:

aligned_seq.append(sequence[0])

reference_seq.append(sequence[1])

seq_frequency.append(sequence[9])

return aligned_seq, reference_seq, seq_frequency

def get_index(position,ref_sequence):

'''

will identify the index in the reference sequence

that corresponds what would be the index if the reference

sequence did not have any insertions

argument:

position = any integer

ref_sequence = the 'reference sequence' of an allele

returns

index : the index of ref_sequence that corresponds

what would be the index if the reference

sequence did not have any insertions

'''

#loop to get index and count:

#index keeps track of ref_sequence index, and

# count keeps track of what would be the index if the

# reference sequence did not have any insertions

index = 0

count = 0

while count < int(position) and index <= len(ref_sequence):

if ref_sequence[index] != '-':

count += 1

index += 1

#returns index

return index

def make_row(trim_align, trim_ref): #makes the formated output row

'''

makes the formated output row with

deletions = '-'

insertions = 'lower case letter (base)' on index before insertion

mutation = Capital letter (base) that mutation changed from

(base of reference sequence)

argument:

trim_align = "aligned sequence' that has been trimed to

user declared indexes

trim_ref = "reference sequence' that has been trimed to

user declared indexes

'''

#loop to make formated output row indicating

# insertions, deletions, and mutations

ref_index = 0

outrow = []

for i, base in enumerate(trim_ref):

#follow path if there are no insertions

if trim_ref[i] != '-':

#fallow path if there are NO mutations

if trim_align[i] == trim_ref[i]: #no indel or insertion

outrow.append(' ')

#follow path if there ARE deletions

elif trim_align[i] == '-':

outrow.append('-')

#follow path if there ARE mutations

else:

outrow.append(trim_align[i])

ref_index += 1

else: #follow path if there IS an insertion

if len(outrow)>= 1:

outrow[-1] = outrow[-1] + trim_align[i].lower()

#return formated ouput row as a list

return outrow

def make_table(aligned_list, reference_list,

beg_index, end_index, frequency):

'''

makes formated table using make_row() and get_index()

functions. Assumes that there will be no case in which there

is an insertion at the beggining index. Omitts alleles that

does not have either an aligned or reference sequence

arguments

aligned_list = list of 'aligned sequence' from each row of file

reference_list = list of 'reference sequence' from each row of file

beg_index = user defined (via graphics) begining index

of area of interest

end_index = user defined (via graphics) end index

of area of interest

frequency = list of 'frequency' from each row of file

returns:

table : formated representation of insertions, deletions,

and mutations followed by corresponding allele

frequency, 1 line per allele

'''

#loop to make each row of table

table = []

for i, allele in enumerate(reference_list):

#if both aligned_sequence and reference sequence

# exist for an allele (row of input file),

# make corresponding row in table

if allele != '#NAME?' and aligned_list[i] != '#NAME?':

#finds starting index of reference sequence

start = get_index(beg_index, reference_list[i])

#finds ending index of reference sequence

end = get_index(end_index, reference_list[i])

#trims aliged and reference sequence to equivalent

# of user defined beggining and ending indexes

trim_align = (aligned_list[i][start:end+1])

trim_ref =(reference_list[i][start:end+1])

#make each row of table

if len(trim_align) == len(trim_ref):

#row = make_row(trim_align, trim_ref)

row = make_row(trim_align, trim_ref) + [frequency[i]]

#append row to table list

table.append(row)

return table

def write_file(table, output_name, beg_index,end_index, name_interest):

'''

writes file of table produced in make_table() function, and

its correspoding header

arguments

table = formated table from make_table() function

output_name = user defined (via graphics) name of file

to be written

beg_index = user defined (via graphics) beggining index

of area of interest

end_index = user defined (via graphics) end index

of area of interest

name_interest = user defined (via graphics) name

of area of interest

returns

none

'''

#loop to tab delimit table with insertions, deletions

# and mutations

final_table = []

for i,entry in enumerate(table):

final_table.append(str(i+1) + '\t' +'\t'.join(entry) + '\n')

#opens file to be written as user defined output name

with open((output_name + '.txt'), 'w') as files:

#writes header, with name of region

files.write('The following information is for the' \

+ ' '+ str(name_interest) +' ' + 'region' + '\n')

files.write('Sequence Name' + '\t')

#writes header with corresponding indexes in

# user defined range

for i in range(beg_index, end_index +1):

files.write(str(i) + '\t')

#writes frequency header

files.write('Frequency' + '\n')

#writes everyrow of the final formated table

files.writelines(final_table)

def main():

#calls user_input function and saves

# returned varibales

user_info = user_input()

file_name = user_info[0]

name_interest = user_info[1]

beg_index = user_info[2]

end_index = user_info[3]

output_name = user_info[4]

#calls list_file() function and saves

# returned varibales

list_file = get_file_info(file_name) #works

aligned_list = list_file[0]

reference_list = list_file[1]

frequency= list_file[2]

#calls make_table() function and saves

# returned varibale

table = make_table(aligned_list,

reference_list, beg_index, end_index,frequency)

#calls write_file() function

write_file(table,output_name, beg_index,end_index, name_interest)

#lets user know to check for file in computer

print 'Analysis is done!'

print 'check folder with this code for the excel file with your analysis!'

main()
