# Supplementary material for: Attempted use of PACE for riboswitch discovery generates three new translational theophylline riboswitch side products
Source: BMC Res Notes. 2018 Dec 5;11:861. doi: 10.1186/s13104-018-3965-6 (PMC6280357; doi:10.1186/s13104-018-3965-6)
Supplement: Supplementary file 2 — Additional file 2. Chemostat Manual: user manual for building a chemostat for phage-assisted continuous evolution (PACE). This is a detailed explanation of how to build your own chemostats for PACE experiments. This document also contains all parts numbers and suppliers so users can purchase the same materials we used. [file 13104_2018_3965_MOESM2_ESM.docx]

| Chemostat Manual |
| --- |
| User manual for building a chemostat  for Phage-Assisted Continuous Evolution (PACE) |
|  |
| **Austin Sprague, Missouri Western State University** |
| **Cody Herron, Davidson College**  **August, 2016** |

**Table of Contents**

1. Chemostat Part List
   1. Part List with Supplier Information…………………………………………….Page 1
2. Building the Chemostat
   1. Lagoons and Host………………………………………………..……………………Page 2
   2. Peristaltic Pump Assembly……………………………………………..…………Page 3
   3. Connecting Tubing for 325mL/hr Pump…………………………………….Page 4
   4. Connecting Tubing for 100mL/hr Pump………………………………..…..Page 6
   5. Connecting Tubing for Syringe Pump…………………………………………Page 7
   6. Final Connections (Sampling Port).......………………………………………Page 7
3. Deconstructing/Reassembling for Autoclaving
   1. Deconstructing Chemostat for Autoclaving…………………….….……..Page 8
   2. Reassembling Chemostat after Autoclaving………………………………Page 9
4. Calibrating and Troubleshooting the Chemostat
   1. Calibrating the Chemostat…………………………………………………........Page 11
   2. Troubleshooting The Chemostat……………………………………………….Page 12
   3. Sampling from Lagoons and Host………………………………………..…….Page 12
   4. Operating Temperatures and Stir Speed……………………………………Page 12

| **Item** | **Use** | **Supplier and Item Number** | **Unit Price** | **Package Quantity** | **Amount Needed** | **Autoclavable?** |
| --- | --- | --- | --- | --- | --- | --- |
| Hot Plate | Temperature Control | Benchmark H3760-HS | $195 | 1 Hot Plate | 4 Hot Plate | No |
| Syringe Pump | Inducer Pump | Cole Parmer  75900-50 | $2475 | 1 Pump | 1 Pump | No |
| Peristaltic Pump Driver | Fluid Pump | Cole Parmer  07554-80 | $706 Per Pump Drive | 1 Pump Driver | 2 Pump Drivers | No |
| Peristaltic Pump Head | Fluid Pump | Cole Parmer  07519-05 | $479 Per Pump Head | 1 Pump Driver | 2 Pump Heads | N/A |
| Peristaltic Pump Cartridge | Fluid Pump | Cole Parmer  07519-80 | $77 Per Cartridge | 1 Pump Cartridge | 8 Cartridges | N/A |
| 1/16” Male Luer Lock | Tubing Connector | Cole Parmer  45505-00 | $0.39 Per Luer | 25 Luers per pack | 100 Luers | Yes |
| 1/16” Female Luer Lock | Tubing Connector | Cole Parmer  45502-00 | $0.35 Per Luer | 25 Luers per pack | 50 Luers | Yes |
| 1/4” Male Luer Lock | Tubing Connector | Cole Parmer  45505-19 | $0.42 Per Luer | 25 Luers per pack | 25 Luers | Yes |
| 0.035in Extension Tubing | Fluid Transfer | Cole Parmer  95809-26 | $1.27 Per Foot | 100ft spool | 100ft | Yes, up to 5 times |
| 1.42mm Pump Tubing | Fluid Transfer | Cole Parmer  06421-34 | $13.21 Per Tube | 6 Pack of Tubing | 1 Pack of 6 Tubes | Yes |

| **Item** | **Use** | **Supplier and Item Number** | **Unit Price** | **Package Quantity** | **Amount Needed** | **Autoclavable?** |
| --- | --- | --- | --- | --- | --- | --- |
| 2.06mm Pump Tubing | Fluid Transfer | Cole Parmer  06421-42 | 14.50 Per Tube | 6 Pack of Tubing | 1 Pack of 6 Tubes | Yes |
| 0.25in Extension Tubing | Fluid Transfer | Cole Parmer  95802-12 | 2.61 Per Foot | 25ft spool | 25ft | Yes |
| 100mL Media bottle | Lagoon | Cole Parmer  34514-22 | 8.91 Per Bottle | 10 Bottles Per Case | 1 Case of 10 Bottles | Yes |
| 500mL Media bottle | Host | Cole Parmer  34514-24 | 11.7 Per Bottle | 10 Bottles Per Case | 1 Case of 10 Bottles | Yes |
| G45 Septum | Host/Lagoon Closure | Fisher Scientific  06-414-12 | 9.04 Per Septum | 10 Septum Per Pack | 1 Pack of 10 Septum | Yes |
| G45 Jar Cap | Host/Lagoon Closure | Fisher Scientific  06-414-9 | 19.40 Per Cap | 10 Caps Per Pack | 1 Pack of 10 | Yes |
| 20L Carboy | Media | Cole Parmer  62507-20 | 77 Per Carboy | 1 Carboy Per Case | 2 Carboys | Yes |
| 18 Gauge, 1.5in Needle (Disposable) | Fluid Addition | VWR  BD305180 | 0.34 Per Needle | 100 Needles Per Pack | 2 Packs of 100 Needles | **NO** |
| 18 Gauge, 6” Long Needle | Fluid Withdrawal | VWR  20068-682 | 13.18 Per Needle | 12 Needles Per Pack | 1 Pack of 12 Needles | Yes |
| 14 Gauge Needle | Sterile Air Flow | VWR  89220-914 | 29.33 Per Needle | 6 Needles Per Pack | 1 Pack of 6 Needles | Yes |
| Large Air Filter | Sterile Air Flow | VWR  28137-652 | 11.73 Per Filter | 10 Filters Per Pack | 1 Pack of 10 Filters | Yes |
| 0.2um Air Filter | Sterile Air Flow | VWR  28137-650 | 4.63 Per Filter | 50 Filters Per Pack | 1 Pack of 50 Filters | Yes |
| 60mL Syringe | Inducer | VWR  BD309653 | 1.46 Per Syringe | 40 Per Pack | 2 Packs of 40 Syringes | **NO** |
| Chemical Tray | Spill/Hazard Prevention | Cole Parmer  06715-90 | 50.4 Per Tray | 1 Per Case | 1 Tray | N/A |

**Building the Chemostat**

1. Lagoons and Host
2. Clear enough room so that the Chemical Tray, 2 Peristaltic Pumps, 1 Syringe Pump, and 1 Carboy can comfortably sit with a minimum of 3in between each item. *NOTE: You will need access to a minimum of 7 power outlets, so a power strip is recommended.*
3. Place the Chemical Tray so that the longer sides are parallel with the surface edge.
4. Place 4 Hot Plates side by side inside the Chemical Tray keeping a minimum of 3in between each hotplate and the walls of the Chemical Tray.
5. Acquire 3 100mL bottles, 1 500mL Bottle, and 4 Magnetic Stir Bars. Place the 500mL bottle on the far right Hot Plate, and the 100mL bottles on the remaining 3 Hot Plates. Label from right to left: “Host”, “Lagoon 1”, “Lagoon 2”, and “Lagoon 3”. Place 1 Magnetic Stir Bar in each Bottle.
6. Acquire 4 G45 Septums and 4 G45 Caps. Place 1 Septum in each Cap and push the Septum up to the top of the Cap until the Septum is firm against the retainer.
7. Firmly screw the Cap and Septum onto a Bottle (it does not matter which bottle type gets what Cap and Septum).
8. For each 100mL bottle acquire: 1 18 Gauge 6” Needle, 1 14 Gauge Needle, and 1 0.2um air filter, and acquire an extra 3 18 Gauge 6” Needle for the 500mL bottle.
9. Place each needle in the bottle by piercing the Septum so that the 6” Needles are 75% in the bottle and 25% out, and the 14 Gauge Needle is as far in as reasonably possible.
10. Attach the 0.2um Filter to the opening of the 14 Gauge Needle.
11. Acquire a block of Styrofoam that is 1in wider in all directions than the 100mL Bottles used for the Lagoons, and is as tall as from the bottom of the Bottle to where the Lid meets the glass.
12. Hollow out the Styrofoam so that the Lagoon Bottle fits tightly inside.
13. Cut out a roughly 2in wide by 1in tall viewing port into one side of the Styrofoam where the 40mL mark on the bottle would be.
14. Orient the Lagoon Bottle so that the volume marks are visible through the viewing port.
15. Repeat steps 10-13 for the other 2 Lagoons.
16. Peristaltic Pump Assembly
17. Acquire 2 Peristaltic Pump Drivers, 2 Peristaltic Pump Heads, 8 Peristaltic Pump Cartridges, 5 2.06mm Pump Tubing, and 3 1.42mm Pump Tubing.
18. Follow the Manufacturer’s Instructions to assemble the Peristaltic Pump Drivers and Heads.
19. Place the 2 Peristaltic Pumps side by side – with a small gap between – or one on top of the other if space is an issue (being sure to use the adjustable foot on the Pump Head if needed).
20. Attach 1 Pump Tubing per 1 Cartridge. Then group them based on Tubing size.
21. Place a strip of masking tape on the top of each Cartridge.
22. Designate 1 Peristaltic Pump to be for a flow rate of 325mL/hr, and the other to be for a flow rate of 100mL/hr.
23. Attach the Cartridges to the Peristaltic Pump Heads:

Use the Cartridges with the 2.06mm Tubing for the Pump designated for 325mL/hr and the Cartridges with the 1.42mm Tubing for the Pump designated for 100mL/hr. *NOTE: Tubing size can be determined by the color of the plastic attached to the tubing, where purple signifies 2.06mm ID and orange signifies 1.42mm ID.*

To attach a Cartridge to the Pump Head, orient the Cartridge so that the Occlusion Adjuster is on the right. Then rotate the Cartridge roughly 90° clockwise and align the semicircular groove on the cartridge with the bar on the Pump Head. Push until the bar on the Pump Head is in the Cartridge groove.

Being sure to keep the bar on the Pump Head in the Cartridge Groove, rotate the Cartridge counter-clockwise until the clip on the left of the Cartridge snaps onto the left Pump Head bar. *NOTE: Addition force may need to be used on the Cartridge clip to insure that the clip is properly seated on the Pump Head bar.*

Repeat steps ii and iii for each respective Cartridge and Pump. To prevent unwanted motion of the Cartridges, slide the o-ring located on each Pump Head bar toward the Cartridges until the o-rings are flush with the Cartridges.

1. Now that the Cartridges are secured on their respective Pump Heads (2.06mm to 325mL/hr Pump and 1.42mm to 100mL/hr Pump), take a fine tip sharpie and write on the masking tape previously placed on the Cartridges.

The most important thing to remember is to be consistent with naming, so it is recommended to use “H” to represent Host, “Ln” to represent Lagoon (where n is the Lagoon number), “M” to represent Media, and “W” to represent waste.

Following this naming pattern, label the Cartridge the farthest away from you on the Pump Designated for 325mL/hr, “M to H”, to represent the transfer of Media to Host.

Moving toward you, label the next Cartridge, “H to W”, to represent the transfer of Host fluid to Waste.

Continue to move toward you. Label the next Cartridge, “L1 to W”, to represent the transfer of Lagoon 1 fluid to Waste.

Label the next Cartridge, “L2 to W”, to represent the transfer of Lagoon 2 fluids to Waste.

Label the final Cartridge, “L3 to W”, to represent the transfer of Lagoon 3 fluids to Waste.

Now move onto the Pump designated for 100 mL/hr; again, starting with the Cartridge farthest from you, label it “H to L1”, to represent the transfer of Host fluids to Lagoon 1.

Moving toward you, label the next cartridge, “H to L2”, to represent the transfer of Host fluids to Lagoon 2.

Label the last Cartridge “H to L3”, to represent the transfer of Host fluids to Lagoon 3.

1. Connecting Tubing for 325mL/hr Pump

*NOTE:* An important fact to remember when connecting tubing is that the direction of flow is counter-clockwise, or from the right side of the Cartridge to the left. Also, be sure that all Tubing is unobstructed and is not being pinched by other objects.

1. Start by acquiring a Carboy Lid, 1/4" Extension Tubing, 1/4" Male Luer Locks, and a Large Air Filter.
2. Attach a 1/16” Male Luer Lock to each end of the 2.06mm Pump Tubing on each Cartridge for the 325mL/hr Pump. To do this, insert the barbed end on the Male Luer Lock into the end of the Tubing until the end of the Tubing is past the barb.
3. Cut off about 4in of 1/4" Extension Tubing, and attach one end of the Tubing to one of the barbs on the Carboy Lid, and the other end to the Large Air Filter.
4. Flip the Lid over so that the underside is facing towards you. Make sure that more 1/4” Tubing (roughly 6in) is connected to the barb that connects to the Large Air Filter on the other side of the Lid.
5. Acquire 0.89mm (0.035in) extension tubing, 1/16” Male Luer Locks, 1/16” Female Luer Locks, and 1.5in Disposable Needles.
6. Place a Carboy designated for the Waste in the spot where it is going to stay during experimentation.
7. Measure out the length of Extension Tubing needed to make it from the Peristaltic Pump designated for 325mL/hr to the Waste Carboy (remember that the Extension Tubing needs to reach the **LEFT** side of the Cartridge), making sure the Tubing will stay in the Waste Carboy (at least 9in of Tubing inside the Carboy is recommended).
8. Now cut 4 pieces of Extension Tubing the length determined in step 6.
9. Now attach a 1/16” Female Luer Lock onto ONE end of each piece of Extension Tubing that was cut in step 8 by inserting the barbed end of the Female Luer Lock into the end of the Extension Tubing, making sure that the end of the Extension Tubing is past the barb.
10. Now connect the Female Luer Locks of the Extension Tubing to the Male Luer Locks on the **LEFT** side of the 2.06mm Pump Tubing in the Cartridges labeled, “H to W”, “L1 to W”, “L2 to W”, and “L3 to W”.
11. Place a 1/16” Male Luer Lock onto the other end of each Extension Tubing, and place these ends into the Waste Carboy for now.
12. Place the Media Carboy in the Spot that it will be when running experiments.
13. Measure out, and cut, a length of 0.89mm (0.035in) Extension Tubing from the **RIGHT** side of the Cartridges with 2.06mm Pump Tubing to the **BOTTOM** of the Media Carboy.
14. Attach a 1/16” Female Luer Lock to one end of the 0.89mm (0.035in) Extension Tubing, and then snake the other end through one of the barbs on the top of the Carboy Lid. Then attach a 1/16” Male Luer Lock to the other end.
15. Connect the Male Luer Lock on the **RIGHT** side of the Cartridge labeled “M to H” to the Female Luer Lock on the Extension Tubing previously cut in step 13. For now, place the other end into the Media Carboy.
16. Measure out and cut a piece of 0.89mm (0.035in) Extension Tubing from the **LEFT** side of the Cartridge labeled “M to H” to the top of one of the 6in Needles in the Host Bottle (leave some slack in the Extension Tubing so that it is not pulling too much on the 6in Needle).
17. Attach a 1/16” Male Luer Lock to one end of the Extension Tubing and a 1/16” Female Luer Lock to the other end.
18. Connect the Female Luer Lock on the Extension Tubing to the Male Luer Lock on the **LEFT** side of the Cartridge Labeled “M to H”. Then, connect the Male Luer Lock on the Extension Tubing to a 1.5in Disposable Needle and Pierce the Septum of the Host Bottle.
19. Measure out and cut a piece of 0.89mm (0.035in) Extension Tubing from the **RIGHT** side of the Cartridge labeled “H to W” to one of the 6in Needles in the Host Bottle. Be sure to leave slack so that the Extension Tubing is not being pulled or stretched.
20. Attach a 1/16” Male Luer Lock to one end of the Extension Tubing, and a 1/16” Female Luer Lock to the other end.
21. Attach the Female Luer Lock on the Extension Tubing to the Male Luer Lock on the **RIGHT** side of Cartridge labeled “H to W”, and the Male Luer Lock on the Extension Tubing to one of the 6in Needles in the Host Bottle.
22. Measure and cut a piece of 0.89mm (0.035in) Extension Tubing from the **RIGHT** side of the Cartridge labeled “L1 to W” to the 6in Needle in the L1 Bottle. Be sure to leave enough slack in the Extension Tubing so that the Tubing is not being pulled or stretched.
23. Attach a 1/16” Male Luer Lock to one end of the Extension Tubing, and a 1/16” Female Luer Lock to the other end of the Extension Tubing.
24. Connect the Female Luer Lock on the Extension Tubing to the **RIGHT** side of the Cartridge labeled “L1 to W”, and the Male Luer Lock on the Extension Tubing to the 6in Needle in the L2 Bottle.
25. Measure and cut a piece of 0.89mm (0.035in) Extension Tubing from the **RIGHT** side of the Cartridge labeled “L2 to W” to the 6in Needle in the L2 Bottle. Be sure to leave enough slack in the Extension Tubing so that it is not being pulled or stretched.
26. Attach a 1/16” Male Luer Lock to one end of the Extension Tubing and a 1/16” Female Luer Lock to the other end of the Extension Tubing.
27. Connect the Female Luer Lock on the Extension Tubing to the **RIGHT** side of the Cartridge labeled “L2 to W”, and the Male Luer Lock on the Extension Tubing to the 6in Needle in the L2 Bottle.
28. Measure and cut a piece of 0.89mm (0.035in) Extension Tubing from the **RIGHT** side of the Cartridge labeled “L3 to W” to the 6in Needle in the L3 Bottle. Be sure to leave enough slack in the Extension Tubing so that it is not being pulled or stretched.
29. Attach a 1/16” Male Luer Lock to one end of the Extension Tubing and a 1/16” Female Luer Lock to the other end of the Extension Tubing.
30. Connect the Female Luer Lock on the Extension Tubing to the RIGHT side of the Cartridge labeled “L3 to W”, and the Male Luer Lock on the Extension Tubing to the 6in Needle in the L3 Bottle.
31. Connecting Tubing for 100mL/hr Pump

*NOTE:* An important fact to remember when connecting tubing is that the direction of flow is counter-clockwise, or from the right side of the Cartridge to the left. Also, be sure that all Tubing is unobstructed and is not being pinched by other objects.

1. Connect a 1/16” Male Luer Lock to each end of the 1.42mm Pump Tubing in the Cartridges for the 100mL/hr Pump.
2. Measure and cut 3 pieces of 0.89mm (0.035in) Extension Tubing from the **RIGHT** side of the Cartridges on the Pump designated for 100mL/hr to the other 3 6in Needles in the Host Bottle.
3. Connect a 1/16” Male Luer Lock to one end of each of the pieces of Extension Tubing, and a 1/16” Female Luer Lock to the other end of each of the pieces of Extension Tubing.
4. Connect the Female Luer Lock on each piece of Extension Tubing to the **RIGHT** side of the Cartridges marked “H to L1”, “H to L2”, and “H to L3”. Then, connect the Male Luer Lock on each of the Extension Tubing to a 6in Needle in the Host Bottle.
5. Measure and cut a piece of 0.89mm (0.035in) Extension Tubing from the **LEFT** side of the Cartridge labeled “H to L1” to the top of the L1 Bottle. Be sure to leave enough slack in the Extension Tubing so that the Extension Tubing is not being pulled or stretched.
6. Attach a 1/16” Male Luer Lock to one end of the Extension Tubing, and a 1/16” Female Luer Lock to the other end of the Extension Tubing.
7. Connect the Female Luer Lock on the Extension Tubing to the **LEFT** side of the Cartridge labeled “H to L1”, and the Male Luer Lock on the Extension Tubing to a 1.5in Disposable Needle. Penetrate the Septum of L1 with the Disposable Needle.
8. Measure and cut a piece of 0.89mm (0.035in) Extension Tubing from the **LEFT** side of the Cartridge labeled “H to L2” to the top of the L2 Bottle. Be sure to leave enough slack in the Extension Tubing so that the Extension Tubing is not being pulled or stretched.
9. Attach a 1/16” Male Luer Lock to one end of the Extension Tubing, and a 1/16” Female Luer Lock to the other end of the Extension Tubing.
10. Connect the Female Luer Lock on the Extension Tubing to the **LEFT** side of the Cartridge labeled “H to L2”, and the Male Luer Lock on the Extension Tubing to a 1.5in Disposable Needle. Penetrate the Septum of L2 with the Disposable Needle.
11. Measure and cut a piece of 0.89mm (0.035in) Extension Tubing from the **LEFT** side of the Cartridge labeled “H to L3”to the top of the L3 Bottle. Be sure to leave enough slack in the Extension Tubing so that the Extension Tubing is not being pulled or stretched.
12. Attach a 1/16” Male Luer Lock to one end of the Extension Tubing, and a 1/16” Female Luer Lock to the other end of the Extension Tubing.
13. Connect the Female Luer Lock on the Extension Tubing to the **LEFT** side of the Cartridge labeled “H to L3”, and the Male Luer Lock on the Extension Tubing to a 1.5in Disposable Needle. Penetrate the Septum of L3 with the Disposable Needle.
14. Connecting Tubing for Syringe Pump

*NOTE:* Consult the Syringe Pump Manual for operation, and for instructions on placing Syringes in the Syringe Pump.

1. Acquire 0.89mm (0.035in) Extension Tubing, 1.5in Disposable Needles, 1/16” Female Luer Locks, 1/16” Male Luer Locks, and 60mL Syringes.
2. Measure and cut a piece of 0.89mm (0.035in) Extension Tubing from the top of the Syringe Pump (to the middle is recommended) to the top of the L1 Bottle.
3. Attach a 1/16” Male Luer Lock to one end of the Extension Tubing, and a 1/16” Female Luer Lock to the other end of the Extension Tubing.
4. Connect the Female Luer Lock on the Extension Tubing to the 60mL Syringe, and the Male Luer Lock to 1.5in Disposable Need. Penetrate the Septum of the L1 Bottle with the Disposable Needle.
5. Repeat steps 2 through 4 for L2 and L3.
6. Final Connections (Sample Port)
7. Screw the Carboy Lid onto the Media Carboy being sure not to pull on the Extension Tubing that has been snaked through it.
8. Connect a 1/16” Male Luer Lock to a 1/16” female Luer Lock.
9. Find where the Extension Tubing come out the top of the Carboy Lid and cut the Extension Tubing around there, being sure to hold on to both ends.
10. Place the Male/Female Luer Lock from step 2 between the two pieces of Extension Tubing so that the Male end is facing toward the Peristaltic Pump, and the Female end is facing toward the Carboy Lid.
11. Push the 6in Needle in each Lagoon down to where the end is touching the 40mL mark on the Bottle. Push one of the 6in Needles in the Host Lid so that the tip is roughly at the 250mL mark on the Bottle, and push the other 6in Needles completely in.

**Deconstructing/Reassembling Chemostat for Autoclaving**

1. Deconstruction of Chemostat for Autoclaving

*NOTE:* Cleaning out the lines of the system after each experiment is recommended to avoid clogging the lines. To do this, empty each Lagoon and Host of previous contents, and add clean water so that all of the 6 in needles are submerged. Unscrew the Carboy Lid and place the end in a Beaker with clean water. Run the Chemostat for about 5 minutes. Then change out the water in the Lagoons and Host, and run the Chemostat for another 5 minutes. Finally, empty out all Lagoons, Host, and the Beaker that the Carboy Lid end was in. Run the Chemostat for 1 minute to get the water out, and empty the Lagoons and Host of any remaining water. Leave the Stir Bars in their respective Bottles.

1. Acquire 3 500mL Beakers and 1 1L Beaker. Label the 3 500mL Beakers “L1”, “L2”, and “L3” in no particular order. Label the 1L Beaker “H”.
2. Find all of the Extension Tubing with a Disposable Needle attached (the Extension Tubing in the Waste Carboy should be included in this), and make a marking on the Extension Tubing close to where the Disposable Needle was attached. Using a non-black dark colored Sharpie is recommended for easy visibility.
3. Starting with Lagoon 3, remove the Disposable Needles that are piercing the Septum (should be 2) and remove the Disposable Needles from their respective Male Luer Locks. Then place the Lid in the 500mL Beaker labeled “L3”. It is recommended that you tighten all Luer Connections EXCEPT the ones that include non-autoclavable parts.
4. Follow the Extension Tubing running from the 6in Needle of Lagoon 3 to the Cartridge labeled “L3 to W”. Keeping all of the Tubing connected, remove the Pump Tubing from the Cartridge.
5. Remove any Disposable Needles and IV Ports that are along the Tubing line. Starting at the end of the Tubing that was in the Waste Carboy, roll up the Tubing in a spool with roughly a 3-4in diameter until you reach the Lid of Lagoon 3 inside the 500mL Beaker. Tuck the spool of Tubing beside the Lid without pulling on the 6in Needle.
6. Find the Extension Tubing from the Syringe Pump to Lagoon 3 and disconnect the 60mL Syringe. Roll the Extension Tubing into a spool with roughly a 3-4in diameter and place beside the Lagoon 3 Lid in the 500mL Beaker. Wrap the top of the 500mL Beaker and the Lagoon 3 Bottle in Foil for Autoclaving.
7. Repeat Steps 3-6 for the other 2 Lagoons.
8. For the Host, remove the Disposable Needle and then remove the Disposable Needle from its respective Male Luer Lock. Place aside for now.
9. Place the Host Lid into the 1L Beaker. It is recommended that you tighten all Luer Connections along the Tubing lines EXCEPT the ones that include non-autoclavable parts.
10. Remove the 3 lines of Tubing that go through the 100mL/hr Pump and roll them up into a spool roughly 3-4in in diameter starting at the ends. Place the spools in 1L Beaker beside the Host Lid.
11. Remove the Pump Tubing from the Cartridge labeled “H to W”, and roll the Tubing into a spool roughly 3-4in in diameter starting at the end. Place the spool in the 1L Beaker beside the Host Lid.
12. Find the spot on the Extension Tubing near the Carboy Lid where there is a Male/Female Luer Lock connection. Unscrew this connection and leave the Female Luer Lock side of the Extension Tubing (along with the Carboy Lid) aside for now. Remove the Pump Tubing from the Cartridge labeled “M to H” and roll the Tubing into a spool roughly 3-4in in diameter starting at one end. Place the spool in the 1L Beaker, next to the Host Lid.
13. Cover the Host Bottle and the 1L Beaker in foil.
14. Cover the Female Luer Lock and the barb on the Carboy Lid that it passes through in foil. On the bottom side of the Lid roll the 1/4" Extension tubing up into the Lid.
15. Remove the Disposable Needle from the 0.89mm (0.035in) Extension Tubing and mark the end of the Tubing the same as the other Disposable Needles, but keep the Male Luer Lock attached to the Tubing. Roll the Tubing up into the Lid and cover the bottom of the Lid with foil.
16. Reassembling Chemostat after Autoclaving

*NOTE:* The focus of this section will be to reassemble the Chemostat in a sterile way after autoclaving. To do this, at least 2 people are needed, but 3 people are recommended for reassembling the Host (step 2). It is going to be assumed that not only are you wearing gloves when appropriate, but that gloves are being switched out when appropriate.

1. Place the 500mL Bottle on the right most Hot Plate, the 1L Beaker holding the Host near the Hot Plate, and the Carboy Lid near the 1L Beaker holding the Host. Have 4 IV ports and 5 1.5in Disposable Needles ready.
2. IF TWO PEOPLE: Have Person 1 with one hand remove the foil on the 500mL Bottle, and with the other hand take the Host Lid out of the Beaker and screw the Host Lid onto the 500mL Bottle (does not need to be tight yet, just started). Person 2 will remove the foil from the 1L Beaker with one hand, and with the other they will remove the Tubing that is attached to the Host Lid from the 1L Beaker. Leave the loose Tubing in the 1L Beaker and recover with the foil. Person 1 that screwed the Host Lid onto the 500mL Bottle will now attach a 1.5in Disposable Needle onto the marked Male Luer Locks (be sure to leave the caps on the 1.5in Disposable Needles). Now finish screwing the Host Lid onto the 500mL Bottle. Next, have Person 1 take the foil off of the 1L Beaker. Then Person 2 removes the Tubing that was left in the 1L Beaker. Then Person 1 attaches a 1.5in Disposable Needle to the appropriate Male Luer Lock (be sure to leave the cap on the 1.5in Disposable Needle), and then attaches the other Male Luer Lock to the Female Luer Lock covered in foil on the Carboy Lid. Then wrap the foil that was covering the Female Luer Lock around the barb that the 0.89mm (0.35in) Extension Tubing is snaked through.
3. IF THREE PEOPLE: Have Person 1 with one hand remove the foil on the 500mL Bottle, aand with the other hand take the Host Lid out of the Beaker and screw the Host lid onto the 500mL Bottle (can fully tighten the Host Lid). Person 2 will remove the foil from the 1L Beaker with one hand, and with the other they will remove the Tubing that is attached to the Host Lid from the 1L Beaker. Leave the loose Tubing in the 1L Beaker and recover with foil. Person 3 will attach a 1.5in Disposable Needle onto the marked Male Luer Locks (be sure to leave the caps on the 1.5in Disposable Needles). Next, have Person 1 take the foil off of the 1L Beaker. Then Person 2 will remove the Tubing that was left in the 1L Beaker. Person 3 now attaches a 1.5in Disposable Needle to the marked Male Luer Lock (be sure to leave the cap on the 1.5in Disposable Needle), and then attaches the other Male Luer Lock to the Female Luer Lock covered in foil on the Carboy Lid. Then wrap the foil that was covering the female Luer Lock around the barb that the 0.89mm (0.035in) Extension Tubing is snaked through.
4. Now connect the Pump Tubing to their respective Cartridges using the length of the Tubing as a guideline to which Lagoon it goes to, as well as subsections 3 and 4 of “Building the Chemostat” for guidance on attaching Pump Tubing to Cartridges. Remember that the purple plastic pieces represent the 2.06mm Pump Tubing (325mL/hr Pump) and the orange represents the 1.42mm Pump Tubing (100mL/hr Pump).
5. Follow the Tubing from the **LEFT** side of the Cartridge labeled “M to H” to the 1.5in Disposable Needle at the end of the tubing. Penetrate the Septum of the Host Lid with the 1.5in Disposable Needle.
6. Place one of the 100mL Bottles on the Hot Plate to the left of the Host and label “L1” for Lagoon 1. Then find the 500mL Beaker labeled “L1” and place near the Hot Plate with Lagoon 1. Prepare 2 1.5in Disposable Needles and 1 60mL syringe.
7. Have Person 1 will remove the foil on the 500mL Beaker and remove the Tubing attached to the L1 Lid (Leave the loose Tubing in the 500mL Beaker and recover with foil). Person 2 will remove the L1 Lid and remove the foil covering the L1 Bottle on the Hot Plate, then screw the L1 Lid onto the L1 Bottle (does not need to be tight, just started). Person 2 will then attach a 1.5in Disposable Needle to the marked Male Luer Lock (be sure to leave the cap on the 1.5in Disposable Needle). Finish screwing on the L1 Lid to the L1 Bottle. Place the end of the Tubing with the 1.5in Disposable Needle into the Waste Carboy.
8. Remove the foil covering the 500mL Beaker. Have Person 1 remove the Tubing from the Beaker and attach a 1.5in Disposable Needle to the marked Male Luer Lock. Person 2 will attach a 60mL Syringe to the other end. Now penetrate the Septum of L1 with the 1.5in Disposable Needle.
9. Repeat steps 5-7 for Lagoons 2 and 3.
10. Follow the Tubing from the Cartridge labeled “H to L1” to the 1.5in Disposable Needle at the end of the Tubing. Penetrate the Septum of the L1 Lid with the 1.5in Disposable Needle.
11. Repeat step 9 for Cartridges “H to L2” and “H to L3”.
12. Push the 6in Needle in each Lagoon down to where the end is touching the 40mL mark on the Bottle. Push one of the 6in Needles in the Host Lid so that the tip is roughly at the 250mL mark on the Bottle, and push the other 6in Needles completely in.

**Calibrating and Troubleshooting the Chemostat**

1. Calibrating the chemostat

After building the chemostat, calibration is needed to find the right dial speed that will produce the desired flow rate. This will detail the steps involved along with some recommendations.

1. Calibrating Cartridge Occlusion for 2.06mm Pump Tubing
2. Disconnect the Luer Lock connection near the Media Carboy Lid and place in a 500mL Beaker that is filled with roughly 250mL of water. Remove the 1.5in Disposable Needle from the Host Lid and place in a 100mL Graduated Cylinder.
3. Set the occlusions on all Cartridges to roughly 2, and set the dial speed of the Peristaltic Pump to about 2.
4. Start the Pump. If no water comes out for 15 seconds, then lower the occlusion on the Cartridge labeled “M to H”. Repeat until continuous water flow is achieved. Then continue slowly lowering the occlusion every 15 seconds. The occlusion at which the water is traveling the quickest is what is desired (this does not need to be exact and can be measured by eye), so when the flow begins to slow again back off to the previous occlusion setting.
5. Set the rest of the Cartridges with 2.06mm Pump Tubing to the same occlusion as the one found in step 3.
6. Calibrating dial speed that produces the desired flow rate of 325mL/hr
7. Keep the Luer Lock from the connection near the Media Carboy Lid in a 500mL Beaker that is filled with roughly 250mL of water. Keep the 1.5in Disposable Needle from the Host Lid and place in a 100mL Graduated Cylinder.
8. Pick a dial speed of about 2 and run the pump for 5 minutes to measure the flow rate.
9. Adjust the dial speed accordingly and retest for 5 minutes until 325mL/hr is achieved.
10. Move the 1.5in Disposable Needle from the 100mL Graduated Cylinder and place in a 1L Graduated Cylinder.
11. Now run for 1 hour long intervals and adjust until 325mL/hr is achieved.
12. Reconnect the Luer Lock connection near the Media Carboy Lid and penetrate the Host Lid Septum with the 1.5in Disposable Needle.
13. Using a piece of tape, mark on the Peristaltic Pump the dial speed that 325mL/hr was achieved, in case the dial should get bumped or moved.
14. Calibrating Cartridge Occlusion for 1.42mm Pump Tubing as well as Finding the Desired Flow Rate of 100mL/hr
15. Follow the Tubing from the **RIGHT** side of the Cartridges labeled “H to L1”, “H to L2”, and “H to L3” to the Host Bottle. Disconnect the Tubing from the 6in Needles they are connected to and place in a 500mL Beaker filled with roughly 400mL of water. Then place a 100mL Graduated Cylinder in front of each Lagoon. Remove the 1.5in Disposable Needle that is connected to the Peristaltic Pumps from each Lagoon and place them in the Graduated Cylinder that is in front of their Lagoon.
16. Set the occlusion of the Cartridges to about 2 to start, and set the dial speed to about 2.
17. Start the Pump. If no water comes out for 15 seconds, then lower the occlusion on the Cartridges. Repeat until continuous water flow is achieved. Then continue slowly lowering the occlusion every 15 seconds. The occlusion at which the water is traveling the quickest is what is desired (this does not need to be exact and can be measured by eye), so when the flow begins to slow again back off to the previous occlusion setting.
18. The occlusions should all be very similar to each other.
19. Now run the pump for 5 minutes and measure the flow rate.
20. Adjust the dial speed accordingly and retest for 5 minutes until 100mL/hr is achieved. The occlusion can also be adjusted if the Cartridges are at different flow rates.
21. Now run for 1 hour intervals and adjust until 100mL/hr is achieved.
22. Reconnect the Male Luer Locks in the 500mL Beaker to the 6in Needles in the Host Lid, and penetrate the Lagoons with their respective 1.5in Disposable Needle.
23. Using a piece of tape, mark on the Peristaltic Pump the dial speed that 100mL/hr was achieved, in case the dial should get bumped or moved.
24. Troubleshooting the Chemostat
25. If there are bubbles in any of the Tubing lines besides the ones labeled “H to W”, “L1 to W”, “L2 to W”, and “L3 to W”, then make sure that all connections are secure and tight.
26. If there are bubbles being produced from the 6in Needles in either the Lagoons or the Host, then make sure that all connections are secure and tight. If the problem persists, then change out the 6in Needle as that one may be clogged.
27. If you start pumping and one of the Cartridges quits pumping fluid, then first make sure that all connections are secure and tight. If the problem persists, then start by lowering the occlusion slightly, waiting 15 seconds, then slowly tightening the occlusion. Wait 15 seconds between each time you tighten or loosen the occlusion.
28. Sampling from Lagoons and Host
29. Fill a small beaker with dH2O and place it by the taped down waste lines/IV ports.
30. Attach a disposable needle to a 3 mL syringe. Place this beside the small beaker with the cover off (be careful, although this is safer than having the cover placed back on repeatedly).
31. One 3 mL syringe w/disposable needle will be left uncapped (be careful) on/beside a small beaker with dH2O. This syringe and needle will be reused.
32. Rinse out the syringe by withdrawing dH2O a few times from the small beaker.
33. Dip a Kimwipe in ethanol and wipe the IV port for the lagoon waste line.
34. Insert rinsed needle into IV port center as straight as possible. Hold the T-lock piece so that the IV port is pointing upwards.
35. Slowly withdraw ~0.85 mL of waste cells. Air will be absorbed, if unable to withdraw more, turn needle upward, flick the syringe, and pump the air out. Repeat until you have ~0.85 mL of waste cells.
36. Add the ~0.85 mL of waste cells to 0.15 mL (150 uL) of glycerol in an appropriately labeled (time and lagoon) 1.5 mL centrifuge tube. Shake vigorously before placing in the freezer.
37. If necessary repeat steps 25-29 for the remaining lagoons.
38. Operating Temperatures and Stir Speed

*NOTE:* It is recommended that you use a Stir setting that seems reasonable to you. It was found that for 40mL of fluid in a 100mL Bottle 200rpm was desirable; it was also found that for 250mL of fluid in a 500mL Bottle 250rpm was desirable.

1. To achieve the temperature of 37°C in the Lagoons and Host, it was found that a Hot Plate temperature of 43°C for the 3 Lagoon’s Hot Plates and 45°C for the Host Hot Plate is desirable.
2. To find the desired Hot Plate Temperature, fill the respective Bottles with an amount of water that matches the volume of fluid that will be in the Bottle during experimentation (if 40mL of fluid will be in the Lagoons and 250mL of fluid will be in the Host, then fill the Lagoons with 40mL of water and fill the Host with 250mL of water).
3. Turn on the Hot Plates and the Stirrer and place a thermometer in the Lagoons and Host. Cover the tops of the Bottles with foil.
4. Let it run for 1 hour and measure the temperature of each Bottle. Adjust the temperature accordingly.
5. Repeat step 4 until the desired internal temperature of the fluid is achieved.
